# Coinfection of SARS-CoV-2 and influenza A (H3N2) detected in bronchoalveolar lavage fluid of a patient with long COVID using metagenomic next−generation sequencing: a case report

**DOI:** 10.3389/fcimb.2023.1224794

**Published:** 2023-09-01

**Authors:** Xuefei Liang, Qiushi Wang, Jia Liu, Jing Ma, Yajuan Zhang, Meng Wang, Yang Yu, Linlin Wang

**Affiliations:** ^1^ Department of Respiratory and Critical Care Medicine, Sinopharm North Hospital, Baotou, China; ^2^ Infection Business Unit, Tianjin Novogene Med LAB Co., Ltd., Tianjin, China; ^3^ Infection Business Unit, Novogene Co., Ltd., Beijing, China

**Keywords:** metagenomic next-generation sequencing, SARS-CoV-2, H3N2, Coinfection, diagnose, BALF, long COVID

## Abstract

The growing number of long COVID cases has drawn clinical attention to severe acute respiratory syndrome coronavirus 2 (SARS-CoV-2), which has been spreading worldwide since winter 2019. Its symptoms are not limited to fatigue and shortness of breath but also affect daily life. We report the use of metagenomic next-generation sequencing (mNGS) to detect coinfection with SARS-CoV-2 and influenza A virus in a patient with long COVID. The patient was admitted with fever, expectoration, fatigue, and shortness of breath. The PCR test was negative due to possible clearance of SARS-Cov-2 in the upper respiratory tract of patients with long COVID. Other routine microbiological tests were also negative, making the clinical diagnosis difficult. Bronchoalveolar lavage fluid (BALF) samples were tested using mNGS. The patient was diagnosed and treated promptly, recovered quickly, and continued taking azvudine after discharge; his condition was stable. This study illustrates that mNGS may be valuable for the timely diagnosis of patients with long COVID and their mixed infections.

## 1 Introduction

Severe acute respiratory syndrome coronavirus 2 (SARS-CoV-2) is a single-stranded positive-sense RNA virus of the genus β. It was first identified in the winter of 2019 and has caused a global pandemic. Respiratory droplets and close contact with infected individuals are the primary modes of transmission for SARS-CoV-2. The main manifestations in patients are dry throat, sore throat, cough, fever, and other symptoms, sometimes accompanied by muscle pain, loss of sense of smell and taste, nasal congestion, runny nose, diarrhea, and conjunctivitis (Domen et al., 2022; Fang and Liang, 2022). Most patients recover within 2 to 4 weeks. However, acute respiratory distress syndrome, septic shock, difficult-to-correct metabolic acidosis, coagulation dysfunction, and multiple-organ failure can occur rapidly in severe cases. It is more common in older people, people with underlying chronic diseases, late pregnancies, perinatal women, and obese individuals (Wanhella and Fernandez-Patron, 2022; Gao et al., 2021; Zhou et al., 2021). Furthermore, some patients infected with SARS-CoV-2 have persistent symptoms after an acute attack, which may fluctuate or recur over time; this is referred to as long COVID or long-haul COVID (Soriano et al., 2022). Studies have shown that SARS-CoV-2 viral RNA can persist in certain areas of the lungs for a long time in some patients (Bussani et al., 2023).

SARS-CoV-2 and influenza A viruses are similar in transmission and respiratory symptoms but differ in virulence, treatment, and vaccine availability (Abdelrahman et al., 2020). Influenza A virus is a single-stranded, negative-strand, segmented RNA virus belonging to the Orthomyxoviridae family. The virus frequently causes fulminant infections in the spring and autumn. In spring 2023, influenza A virus in China mainly appeared as H1N1 and H3N2 subtypes. In recent years, there have been several cases of coinfection of SARS-CoV-2 and influenza A virus during the epidemic of COVID-19 (Wu et al., 2020; Fahim et al., 2021). However, all of these cases were infected with SARS-CoV-2 for the first time, and there have been no reports of coinfection with SARS-CoV-2 and H3N2 in patients with long COVID.

Virus isolation and culture are the “gold standards “ for diagnosing viral infection. However, owing to its time-consuming nature and strict limitations, a more rapid and accurate test method is required clinically (Liu et al., 2022). Metagenomic next-generation sequencing (mNGS) does not depend on culture and can be directly applied to nucleic acids in various sample types for unbiased sequencing. Therefore, they are suitable for viruses that cannot be cultured, grow slowly, or are difficult to detect using conventional methods because of their low content. Several studies reported that mNGS improves pathogen detection in patients with negative routine test results, mixed infections, or atypical symptoms. Moreover, owing to their high sensitivity and rapid diagnosis, which can guide clinical precision medicine, they have a certain application value in clinical practice (Chen et al., 2021). Herein, we report a case of coinfection with SARS-CoV-2 and H3N2. To our knowledge, this is the first time a long COVID case has been identified using mNGS.

## 2 Case presentation

**Figure 1:**
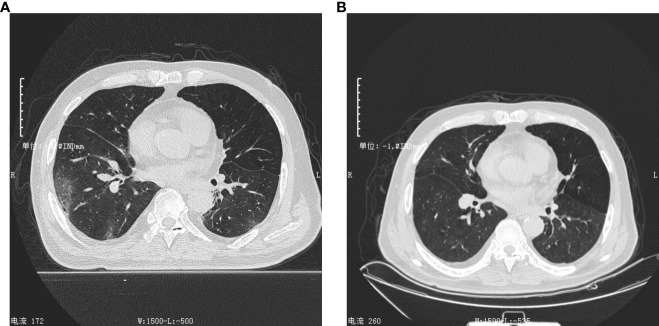
Lung computed tomography (CT) scan. **(A)** CT scan of the first admission on December 20, 2022. This image showed emphysema and multiple ground-glass image changed in both lungs. **(B)** CT scan of the second admission on February 22, 2023. This image showed emphysema, and multiple ground-glass opacities in both lungs were obviously absorbed.

A 73-year-old man from the Inner Mongolia Autonomous Region, China, was admitted to Sinopharm North Hospital (the Third Affiliated Hospital of Baotou Medical College) on February 21, 2023, because of recurrence and aggravation of cough, expectoration, and shortness of breath, accompanied by fever for two months. He had been previously admitted to our hospital for the above symptoms on December 20, 2022. At that time, his body temperature was 38.7°C, he felt weak all over his body, and the nucleic acid test for SARS-CoV-2 was positive. Combined with the results of lung CT (**Figure 1A**), the patient was diagnosed with viral pneumonia. During hospitalization, his condition improved after intravenous injections of sulperazone, levofloxacin, and methylprednisolone, and he was discharged on January 3, 2023. However, one month after his discharge from the hospital, he developed an episodic fever. His body temperature was usually between 37.3°C and 37.5°C. He also had cough, expectoration, and occasionally yellow sputum and felt weak all over the body. On February 22, 2023, his body temperature was higher than normal, reaching 38°C. He visited our hospital for further diagnosis and treatment. Since the onset of the disease, the patient has been experiencing poor mental health, diet, and sleep, but no obvious abnormalities in urine or stool.

The patient had a medical history of underlying chronic disease. Twenty years ago, he developed chest tightness, shortness of breath, cough, and a small amount of white sputum with no apparent cause. The patient had no fever or chest pain. The patient ignored this, which led to recurrent attacks. He was diagnosed with “chronic obstructive pulmonary disease.” He had a history of hypertension for 30 years, and his blood pressure was 180/90 mmHg at one time. He was administered amlodipine (5 mg/day). The patient had a history of diabetes for 30 years. He injected 12 units of subcutaneous insulin glargine every night and took 0.5mg of repaglinide and 25 mg of acarbose tablets every 8 hours to treat hyperglycemia. He has had chronic kidney disease for 4 years. In 2018, he underwent surgical treatment for goiter. Currently, he takes 87.5 ug of levothyroxine sodium tablets per day. In addition, he denied a history of coronary heart disease, “hepatitis, tuberculosis,” and other infections and contact history. He received one dose of COVID-19 vaccine on December 9, 2019, but did not receive the flu vaccine.

On admission, his temperature was 37.3°C, his pulse was 90/min, and his blood pressure was 162/99 mmHg. His blood oxygen saturation was 97%. His mouth was cyanotic, the pharynx was not congested, respiratory sounds were low in both lungs, a little wet and dry rales could be heard in both lungs, the tonsil was not enlarged, heart boundary was not large, heart rate was 90 beats/min, and no obvious pathological murmurs were heard.

**Figure 2:**
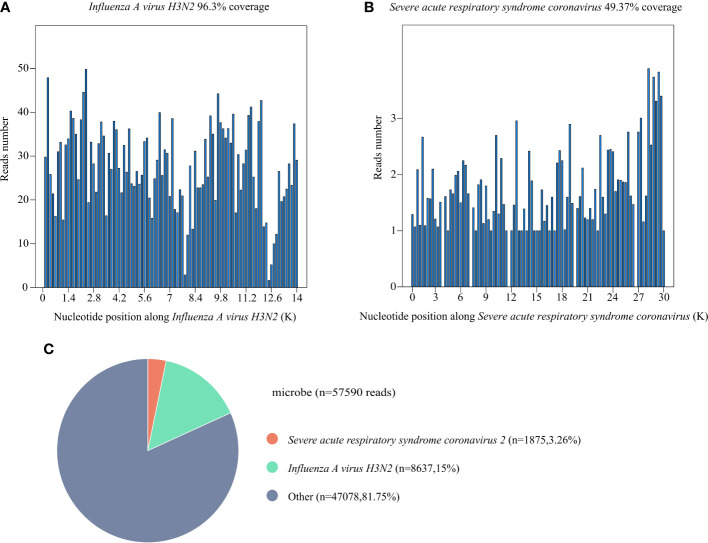
Results of metagenomic next-generation sequencing (mNGS) of bronchoalveolar lavage fluid (BALF) of this patient. **(A)** The coverage of *Influenza A virus H3N2* detected by mNGS. The coverage was 96.3%. **(B)** The coverage of *severe acute respiratory syndrome coronavirus 2 (SARS-CoV-2)* detected by mNGS. The coverage was 49.37%. **(C)** A total of 57590 reads were identified as microbial genome sequences. The percentage of *Influenza A virus H3N2* is 15%, the percentage of SARS-CoV-2 is 3.26%.

**Figure 3:**
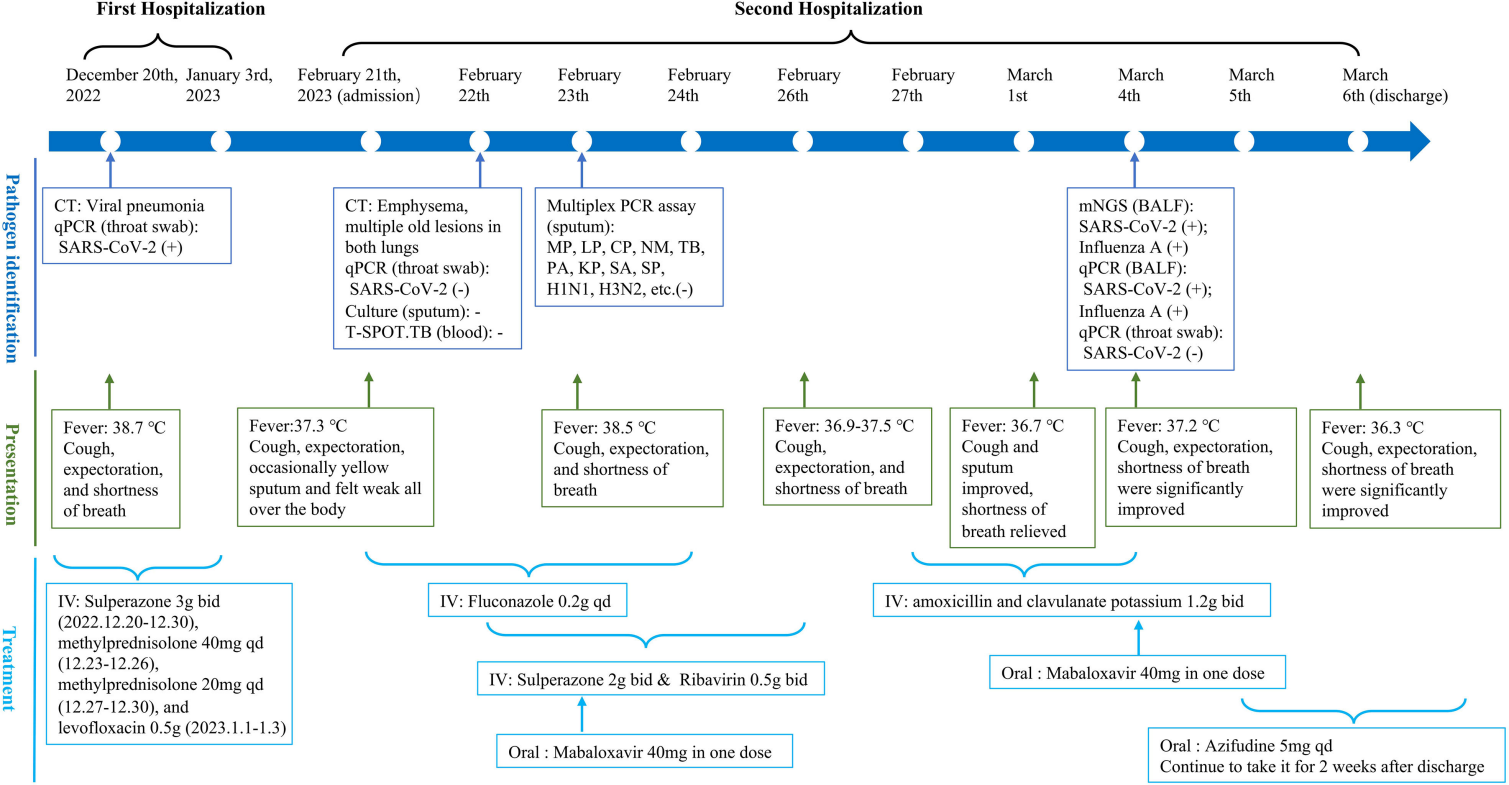
The timeline of the patient for disease course. It shows the process of patients from admission to discharge, mainly including pathogen identification (blue), presentation (green), and treatment (light blue). CT, computed tomography; qPCR, quantitative polymerase chain reaction; SARS-Cov-2, severe acute respiratory syndrome coronavirus 2; TB, *Mycobacterium tuberculosis*; MP, *Mycoplasma pneumoniae*; LP, *Legionella pneumophila*; CP, *Chlamydia pneumoniae*; NM, *Neisseria meningococcus*; PA, *Pseudomonas aeruginosa*; KP, *Klebsiella pneumoniae*; SA, *Staphylococcus aureus*; SP, *Streptococcus pneumoniae*; BALF, bronchoalveolar lavage fluid; IV, intravenous injection.

Laboratory examinations were performed after admission. The items and values used in the examinations are listed in **Table 1**. Leukocyte, neutrophil, monocyte, C-reactive protein, interleukin 6 (IL-6), and procalcitonin (PCT) levels increased while lymphocyte count, CD4 cell count, and CD4/CD8 ratio decreased. Throat swab test results were negative for SARS-CoV-2. We collected sputum for tuberculosis examination, bacterial culture, and DNA amplification for the qualitative detection of pathogens of the respiratory system, including 34 pathogens listed in **Supplementary Table 1**. Pathogen test results were negative. Lung CT showed obvious absorption of the lung infection (**Figure 1B**). The patient was treated with IV fluconazole, sulperazone, ribavirin, and mabaloxavir. His body temperature dropped from the highest 38.3°C to 37.3°C–37.6°C. Subsequently, the antibiotics were changed to amoxicillin and potassium clavulanate, but the low fever persisted. Therefore, a bronchoscopy was performed. Bronchoalveolar lavage fluid (BALF) was collected for bacterial culture and mycobacterium tuberculosis examination, which were negative. At the same time, BALF was sent to Tianjin Novogene Medical Laboratory for mNGS detection. The total number of reads obtained by mNGS was 25356031, and the read length is 50bp. After removing human reads, the remaining reads were 1425503. A total of 57590 reads identified as microbial genome sequences. The mNGS results yielded 18882 sequences for influenza A virus (8637 sequences belonging to H3N2, the rest 10245 sequences of influenza A failed to identify subtypes because they could not be mapped to specific locations of subtypes) and 1875 sequences for SARS-CoV-2 (**Figure 2**). Because BALF is a non-sterile fluid, other pathogens are microecology (listed in **Supplementary Table 2**), which may colonize the respiratory tract and mouth. BALF samples were then tested for influenza A virus (Beijing Applied Biological Technologies Co., Ltd., Beijing, China) and SARS-SARS-CoV-2 (Sansure Biotech Inc., Changsha, China) using commercial PCR-based kits, both of which were positive. On March 4, 40 mg of mabaloxavir was administered orally, but the patient still had a low-grade fever. Blood tests revealed normal liver and kidney functions. On March 5, he was administered oral azvudine 5mg per day for antiviral treatment, and his body temperature normalized. On March 6, his body temperature was normal, and he no longer had a fever. He and his family felt that his shortness of breath and wheezing had improved significantly and that he had no chest pain; therefore, he was discharged from the hospital. After discharge, he was administered oral azvudine 5 mg daily for 2 weeks, and his condition was stable during telephone follow-up. The disease course is shown in **Figure 3**.

**Table 1:** Laboratory test results of the patient.

Items	Reference range	February 22nd	March 5th
White blood cell counts (10^9^/L)	3.5-5.9	10.28	5.46
Neutrophil [%]	40-75	78.3	65.6
Lymphocyte [%]	20-50	9.1	23.3
Monocyte [%]	3-10	10.7	8.8
D-dimer (D-D) (mg/L)	≤1	1.52	1.59
Thyroglobulin antibody (IU/mL)	≤60	75.4	–
Erythrocyte sedimentation rate (MM/H)	0-15	18.64	–
K (mmol/L)	3.5-5.1	3.44	3.72
Urea (mmol/L)	2.8-7.6	5.9	5.1
Creatinine (umol/L)	40-127	105.6	100.87
Uric acid (umol/L)	208-428	381.13	347.28
Alanine aminotransferase (U/L)	10-40	11.05	14.48
γ-glutamyltransferase (U/L)	8-57	17.52	20.08
Total protein (g/L)	68-83	62.28	56.37
Total bile acid (umol/L)	≤13	1.02	3.54
Total bilirubin (umol/L)	5-21	15.36	13.54
C-reactive protein (mg/L)	≤10	43.38	2.15
Interleukin 6 (pg/ml)	<10	39.43	6.08
Procalcitonin (ng/mL)	<0.06	0.28	0.05
CD4 cells/ul	540-1224	480	–
CD8 cells/ul	312-1060	652	–
CD3 cells/ul	1012-2428	1192	–
CD4/CD8 ratio	0.91-2.43	0.74	–

- represents undetected.

## 3 Discussion

This paper reports a case of a patient with long COVID readmitted for fever and other respiratory symptoms 49 days after the initial SARS-CoV-2 positive discharge. After a series of negative microbial tests, influenza A and SARS-CoV-2 were identified in BALF using mNGS. Based on the characteristics of the patient’s symptoms, it is believed that SARS-CoV-2 was responsible for the recurrent fever.

The patient’s clinical symptoms included cough, phlegm, shortness of breath, fever, and malaise. These may be general symptoms of long COVID, which may have new definitions and interpretations as knowledge is updated (Soriano et al., 2022). Currently, there is a study to distinguish prolonged shedding of SARS-CoV-2 viral RNA from true reinfection. This study supports the notion that primary SARS-CoV-2 reinfection is unlikely within 90 days of symptom onset (Nicholson et al., 2021). In this case, symptoms reappeared within 90 days of the onset of primary SARS-CoV-2 infection symptoms; therefore, it can be concluded that the patient was not reinfected. Clinical evidence indicates that patients with diabetes have worse clinical outcomes of COVID-19 than non-diabetic patients (Feldman et al., 2020). This patient had underlying diseases such as diabetes; therefore, potential risks should have been prevented early.

Previous studies have shown that some patients with COVID-19 have decreased white blood cell and lymphocyte counts and increased neutrophil counts, which is consistent with the test results in this case (Huang et al., 2021; Ma et al., 2021). The SARS-Cov-2 inflammatory response may damage lymphoid tissue and increase lymphocyte apoptosis. In addition, erythrocyte sedimentation rate and C-reactive protein, IL-6, and D-dimer levels were elevated. The elevation of these inflammatory markers and abnormal coagulation parameters may be related to the impaired integrity of the epithelial-endothelial barrier and the activation of coagulation (Biamonte et al., 2021). There are various reports on whether CD4 + and CD8 + cells are normal or decreased in patients with COVID-19 (Wang et al., 2020; Wiech et al., 2022). In our case, the CD4+ and CD4+/CD8+ levels were decreased, indicating that the patient’s immunity was weakened. Imaging examinations of the patient in this case showed multiple ground-glass opacity absorptions and transformation into fibrosis in the lower lobes of both lungs, which is consistent with most previously reported patients (Zhao et al., 2020). This indicates that imaging cannot be the sole basis for diagnosing patients with long COVID. In conclusion, reduced lymphocyte count may be important for the diagnosis of long COVID. The increase in inflammatory markers and abnormal coagulation parameters may also be important, with the increase in D-dimer being of great value.

SARS-Cov-2 mixed infections, including combined bacterial, fungal, and viral infections, are common (Sreenath et al., 2021; Mina et al., 2022; Swets et al., 2022). The clinical outcome of respiratory virus coinfection with COVID-19 is currently unclear, but coinfection of COVID-19 with influenza virus and adenovirus is significantly associated with an increased risk of death (Swets et al., 2022). Studies have shown that influenza A infection promotes SARS-CoV-2 infectivity in various cell types and prolongs viral infection in the lungs and bronchoalveolar lavage fluid (BALF), leading to reduced peripheral blood and lymphocytes (Kim et al., 2022). In addition, a decrease in oral microbiome diversity and an increase in the ecological imbalance of species richness have been identified as predictors of COVID-19 (Ren et al., 2021).

Treatment methods for SARS-Cov-2 are mainly divided into antiviral drugs and host-specific therapies. Antiviral drugs mainly include remdesivir, ritonavir, azvudine, and IFN-α, whereas host-specific therapies include neutralizing antibody therapy, Janus kinase inhibitors, and steroids. Several studies have reported that combination therapy has better effects than single therapy (Yuan et al., 2023). The treatment of long COVID mainly focuses on the symptomatic treatment of patients’ symptoms (Chee et al., 2023). However, the antibiotic and antifungal initiation and switching steps were performed without obtaining microbiological results. After detecting the influenza A virus and SARS-Cov-2 using mNGS, the patient was administered abasaclovir, and, after ensuring normal liver and kidney function, azvudine was administered. Baloxavir marboxil is a polymerase inhibitor, and clinical trials have shown that a single dose of baloxavir marboxil can reduce influenza virus titer and disease symptoms. Azvudine, a nucleoside reverse transcriptase inhibitor, shortens the time to turn negative on nucleic acid testing in patients with mild and ordinary COVID-19 and is a relatively safe and effective drug. However, further clinical trials are needed (Kumari et al., 2023).

The patient underwent bronchoscopy. BALF samples were collected for mNGS to detect SARS-CoV-2 and influenza A virus (subtype revealed H3N2). The presence of these two viruses was subsequently verified by detecting BALF using commercially available PCR kits. Influenza A virus was not detected in sputum in the initial hospitalization. The patient was suspected of being infected by his son with influenza A virus during his later hospitalization. However, two throat swab tests during hospitalization were negative for SARS-CoV-2. The sensitivity of SARS-CoV-2 detection in upper respiratory tract specimens may be insufficient (Wikramaratna et al., 2020). Another possibility is that SARS-CoV-2 was shed from the patient’s upper respiratory tract (Ling et al., 2020). Studies have shown that cells expressing the spike or nucleocapsid proteins of SARS-CoV-2 are not found in the respiratory epithelium of patients with COVID-19. However, virus-infected cells are detected in specific areas of the lung, indicating the presence of SARS-CoV-2 RNA (Bussani et al., 2023). Therefore, in cases where SARS-COV-2 infection cannot be ruled out or long COVID is suspected, appropriate BALF samples are required for testing (Chen et al., 2022). mNGS may also be a useful tool for identifying the presence of SARS-CoV-2 in long COVID patients.

The symptoms of long COVID are highly heterogeneous and complex (Fernández-de-Las-Peñas, 2022). Currently, there is no unified standard for the diagnosis and treatment of long COVID, and the long-term pathogenesis of COVID-19 is also unknown (Blomberg et al., 2022). Therefore, it is necessary to accumulate more cases and clinical experience to improve the understanding of long COVID. In addition, further research is needed to investigate the pathological progression in the lungs involving SARS-Cov-2 and influenza A virus and to develop effective epidemiological tracking and diagnosis and treatment programs for long COVID. This study has a suggestive effect on the clinical understanding of symptom characteristics and diagnostic tools for patients with long COVID and their coinfections.

## 4 Conclusion

Patients with long COVID may have been previously overlooked because of improper specimen selection or negative routine tests, especially when coinfection with other viruses is present. This study highlights the value of using mNGS to detect SARS-CoV-2 and influenza A in BALF, which can aid in timely diagnosis. In addition, this method has greater sensitivity and is often used to diagnose fever of unknown origin. Clinicians should be aware of the potential for long COVID in patients with similar characteristics and consider using mNGS to quickly identify and effectively treat infections. Applying mNGS has the potential to greatly benefit patients with long COVID and coinfections.

## Data availability statement

The datasets presented in this study can be found in online repositories. The names of the repository/repositories and accession number(s) can be found below: https://ngdc.cncb.ac.cn/gsa/s/FHmf99y8, CRA011005.

## Ethics statement

The studies involving humans were approved by the Board of Ethics at Sinopharm North Hospital (the Third Affiliated Hospital of Baotou Medical College). The studies were conducted in accordance with the local legislation and institutional requirements. The participants provided their written informed consent to participate in this study. Written informed consent was obtained from the individual(s) for the publication of any potentially identifiable images or data included in this article.

## Author contributions

The study was supervised by YY and LW. XL was involved in the clinical care and management of the patient. The manuscript was written by QW and JL. Clinical data collection and collation were done by XL, JM, and YZ. Sequencing data analysis and data uploading were completed by MW. All authors contributed to the article and approved the submitted version.
