# Supplementary material for: Coinfection of SARS-CoV-2 and influenza A (H3N2) detected in bronchoalveolar lavage fluid of a patient with long COVID using metagenomic next−generation sequencing: a case report
Source: Front Cell Infect Microbiol. 2023 Sep 1;13:1224794. doi: 10.3389/fcimb.2023.1224794 (PMC10505437; doi:10.3389/fcimb.2023.1224794)
Supplement: Supplementary file 1 [file DataSheet_1.pdf]

Supplementary table 1. DNA amplification for the qualitative detection of 34 pathogens of the respiratory system for sputum

Number	Pathogens
1	*Mycoplasma pneumoniae*
2	*Legionella pneumophila*
3	*Chlamydia pneumoniae*
4	*Candida albicans*
5	*Mycobacterium tuberculosis*
6	*influenza A virus*
7	*influenza B virus*
8	*Neisseria meningitidis*
9	*Pseudomonas aeruginosa*
10	*Klebsiella pneumoniae*
11	*Staphylococcus aureus*
12	*Streptococcus pneumoniae*
13	*Haemophilus influenzae*
14	*Adenovirus 1*
15	*Adenovirus 2*
16	*Adenovirus 3*
17	*Adenovirus 4*
18	*Adenovirus 5*
19	*Adenovirus 7*
20	*Adenovirus 14*
21	*Adenovirus 19*
22	*Adenovirus 25*
23	*Acinetobacter baumannii*
24	*Bordetella pertussis*
25	*Escherichia coli*
26	*Human Respiratory syncytial virus*
27	*Human parainfluenza virus*
28	*Rhinovirus*
29	*Human metapneumovirus*
30	*Human gammaherpesvirus 4*
31	*Brucella*
32	*Streptococcus pyogenes*
33	*Streptococcus agalactiae*
34	*Human betaherpesvirus 5*

Supplementary table 2. A list of other microbes that make up 81.75% of reads

**Species name**	**Reads number**
Haemophilus parainfluenzae	10112
Prevotella pallens	6293
Capnocytophaga leadbetteri	6292
Fusobacterium pseudoperiodonticum	5234
Lautropia mirabilis	4556
Mycoplasma orale	3615
Porphyromonas endodontalis	2690
Veillonella atypica	2356
Veillonella parvula	1933
Campylobacter concisus	1787
Neisseria subflava	1022
Peptostreptococcus stomatis	549
Neisseria mucosa	527
Streptococcus mitis	112
